# A Special Section of Articles From the ESF Programme on Functional Genomics Workshop: ‘Data Integration in Functional Genomics and Proteomics: Application to Biological Pathways’

**DOI:** 10.1002/cfg.383

**Published:** 2004-03


					Comparative and Functional Genomics
A Special Section of articles from the ESF Programme on Functional Genomics Workshop
`Data integration in functional genomics and proteomics: application to
biological pathways'
Held at the Swiss Institute of Bioinformatics (SIB), 22-24 September 2003
With editorial assistance from
Pierre-Alain Binz and Paul van der Vet
Workshop Organisers
Pierre-Alain Binz -- Swiss Institute of Bioinformatics (SIB), Switzerland
Paul van der Vet -- Center for Telematics and Information Technology (CTIT), University
of Twente, The Netherlands
Henning Hermjakob -- European Bioinformatics Institute (EBI), UK
The European Science Foundation (ESF) acts as a catalyst for the development of science
by bringing together leading scientists and funding agencies to debate, plan and implement
pan-European scientific and science policy initiatives.
ESF is the European association of 76 major national funding agencies devoted to scientific
research in 29 countries. It represents all scientific disciplines: physical and engineering
sciences, life and environmental sciences, medical sciences, humanities and social sciences.
The Foundation assists its Member Organisations in two main ways. It brings scientists together
in its EUROCORES (ESF Collaborative Research Programmes), Scientific Forward Looks,
Programmes, Networks, Exploratory Workshops and European Research Conferences to work
on topics of common concern including Research Infrastructures. It also conducts the joint
studies of issues of strategic importance in European science policy.
It maintains close relations with other scientific institutions within and outside Europe. By
its activities, the ESF adds value by cooperation and coordination across national frontiers
and endeavours, offers expert scientific advice on strategic issues, and provides the European
forum for science.

				

